# Gender-Based Estimation of Physical Stature Using Percutaneous Lengths of Two Adult Long Bones in a North Indian Population

**DOI:** 10.7759/cureus.99744

**Published:** 2025-12-20

**Authors:** Adhrit Jha, Yuv Yadav, Saroj Sharma, Sushil Kumar, Pratish K Tyagi

**Affiliations:** 1 Department of Anatomy, Amrita School of Medicine, Faridabad, IND; 2 Department of Preventive and Social Medicine, Amrita School of Medicine, Faridabad, IND

**Keywords:** gender, humerus length, north indian population, percutaneous length of long bones, regression equations, stature estimation, tibia length

## Abstract

Background: Accurate estimation of stature from skeletal remains is an essential component of forensic anthropology and medico-legal investigations. For this purpose, long bones are among the most reliable indicators, yet their predictive value varies across populations due to genetic and environmental factors.

Objectives: This cross-sectional observational study was designed using a North Indian adult population to predict stature using long bones (humerus and tibia), individually and in combination, and to compare their predictive accuracy in both genders.

Methods: The study was conducted on 164 healthy adult medical students and working professionals [82 males (M), 82 females (F)], aged 18-55 years at a tertiary care institution, after due institutional ethical clearance. Stature, humerus, and tibia length were measured using standardized anthropometric techniques. Simple linear/multiple linear regression analyses were utilized to predict stature.

Results: The mean stature of all participants was 165.3 ± 8.7 cm, and the humerus length and tibia length for both genders were noted to be 28.6 ± 2.4cm and 38.9 ± 2.6 cm, respectively. Pearson correlation analysis showed a moderate to very strong correlation with humerus (M: r = 0.719, F: r = 0.595) and tibia (M: r = 0.809, F: r = 0.764) length with stature in both genders. In males, the simple linear regression models showed good reliability, with R² values of 0.65 for tibia length and 0.51 for humerus length. The multiple linear regression model using both bone lengths together further improved accuracy, giving an R² of 0.74. In females, humerus (R² = 0.35) and tibia (R² = 0.58) models showed lower reliability, although the combined model produced a better R² of 0.64. The best regression equation to predict stature in males was Stature= 91.32 + 1.34 (tibia) + 0.90 (humerus) at R² = 0.74, whereas in females it was Stature = 67.97 + 1.77 (tibia) + 0.88 (humerus) at R² = 0.646 for the studied population. The study corroborated previous findings that the tibia is a strong predictor and that both bones predict better than a single bone; however, the current values are different from those in previous studies and reflect the changing trends and diversity of the studied population in this region.

Conclusion: Gender-specific regression models enhance precision, as correlations were stronger in males than in females. In both genders, the combined humerus and tibia bones lengths provide a more accurate estimate of stature, and tibia length is a better predictor than humerus length in the case of a single long bone. These findings emphasize the forensic, anthropology, and medical research utility of humerus and tibia measurements in stature estimation within the North Indian population. Further research with other long bone combination and a larger sample size, including higher age groups along with diverse populations, could refine these findings and build upon the results of this study.

## Introduction

Estimation of an individual's stature from skeletal or body measurements is a fundamental aspect of forensic anthropology and its application in various fields. In medico-legal investigations, the stature of an unknown person can be inferred by measuring certain bones or body parts, as many skeletal elements exhibit a strong linear correlation with overall stature [1]. This principle has been successfully applied in identifying victims in mass disasters or other scenarios where only body remains are available [1]. Long bones of the limbs are particularly well known for their stature predictive value, since the classic work of Trotter and Gleser in the mid-20th century [2]. Numerous studies worldwide have derived regression equations linking long bone length to stature [3-6]. However, it is now well established that such equations are population-specific due to genetic, nutritional, and environmental differences between populations [7]. If these equations are applied to another set of populations, they may yield large errors in the estimation of the stature [7], as seen when Pearson’s formula based on Western data was applied on the Indian population [7]. Secular trends and regional variations further necessitate contemporary local data, as formulae from earlier decades have shown underestimation of the stature of the present individuals [8]. This further underlines the importance of deriving updated equations from measurements of the current population [8].

Most studies in India have focused on individual long bones like the femur, tibia, humerus, and ulna [5,9-12], and very few have used multiple long bones [7,13] to formulate a regression equation for stature estimation in different sets of sub-populations. Significant gender differences in regression equations were observed from Indian subjects, suggesting the necessity of gender-based population or sub-group-specific formulae for accuracy [5,7,9,11-13]. Due to a lack of studies involving both genders and multiple bones to address complementary variation in body proportions, secular trends, and regional variations, this study was executed to derive updated equations from measurements. This study includes two easily palpable long bones (tibia and humerus) for percutaneous measurements to study gender-specific stature estimation in the North Indian adult population. We aimed to predict the stature of individuals using simple/multiple linear regression with two long bones, i.e., tibia and humerus, in both genders, and to compare their predictive accuracy.

## Materials and methods

Study design and participants

This cross-sectional observational study was conducted in the Department of Anatomy at a tertiary care institution in Faridabad, Haryana, North India from May 2024 to July 2025. The study was approved by the Institutional Ethics Committee (Reference No.: AIMS-IEC-BAS-04-24-001). The sample size was calculated using Green’s formula [14]: N>50+8p, where N=sample size and p=predictor variables.

Three predictor variables, i.e., length of humerus, tibia, and age, were utilized in Green's formula to estimate the sample size. Hence, for each male and female, the sample size was 74.

A total of 164 adults (82 males and 82 females) were analysed; participation was purely voluntary, and those who consented for participation were measured. The studied population included medical students and working professionals from different states of North India, sharing similar culture and language. The age ranged from 18 to 55 years, representing relatively homogeneous healthy participants. Individuals belonging to other regions, with any history of congenital or acquired bony deformities or significant bone diseases were excluded. Anthropometric measurements and informed written consent were taken from each participant.

Anthropometric measurements

All measurements were carried out using standard anthropometric instruments and protocols [3,11] and were taken by the same observer with the same instrument to avoid any technical and/or inter-observer error. The instruments were regularly checked for their accuracy during data collection. Percutaneous tibia and humerus lengths were chosen to get convenient and accurate measurements in living individuals.

Stature (standing height) was measured to the nearest 0.1 cm using a stadiometer. Each subject stood barefoot on a flat surface with the head positioned in the Frankfurt horizontal plane, the measuring bar was then brought down to touch the vertex of the head. For long bone measurements, percutaneous tibia and humerus lengths were measured using a large sliding caliper [3,11]. The tibia length was taken from the medial condyle of the tibia to the tip of the medial malleolus at the ankle. Percutaneous humerus length was measured from the acromion to the lateral epicondyle by asking the subject to flex the elbow joint at an angle of 90 degrees. The lateral epicondyle was felt, and the acromion point was traced by moving the finger over the clavicle’s lateral end. There was no significant difference observed between right and left side tibia and humerus bone lengths, so all right side bone lengths were recorded. These measurements approximate the maximum length of the tibia and humerus, respectively, via palpable landmarks on the living body, and all were recorded to the nearest 0.1 cm. Each measurement was repeated twice, and the average was used for analysis to ensure intra-observer reliability.

Data analysis

Descriptive statistics (mean, standard deviation, minimum, and maximum values) were calculated for age, stature, humerus length, and tibia length in the total sample and in both genders individually. The distribution of each variable was checked for normality using the Shapiro-Wilk test. Pearson’s correlation coefficient (r) was used to assess the strength of association between stature and three predictor variables. Correlation analysis was performed separately for males, females, and the total participants studied. Based on Evans classification (1996), the interpretation of ‘r’ was as follows: correlations of 0.20-0.39 were considered as weak, 0.40-0.59 as moderate, 0.60-0.79 as strong, and >0.80 as very strong [15]. Prior to regression analysis, data were screened for multicollinearity using Variance Inflation Factors (VIF) and for heteroscedasticity using the Breusch-Pagan test. All assumptions for multiple linear regression were met (VIF < 2.0; Breusch-Pagan p > 0.05). A linear regression equation model was constructed for the parameters that showed significant correlations. A significance level of p < 0.001 was set for all statistical tests. Data compilation and statistical analysis were carried out using Microsoft Excel (Microsoft Corp., Redmond, WA, USA) and JAMOVI software (version 2.7.6), and all results were reviewed for statistical significance.

## Results

A total of 164 adults were included in the study, comprising 82 males (50%) and 82 females (50%). The mean age of the participants was 26.6 ± 9.3 years, with an age range of 18-55 years. The mean stature measured was 165.3 ± 8.7 cm. The mean percutaneous humerus and tibia lengths were 28.6 ± 2.4 cm and 38.9 ± 2.6 cm, respectively (Table 1).

**Table 1:** Age, stature, humerus length, and tibia length of the total study population.

Variable	Mean ± SD	Minimum	Maximum
Age (year)	26.6 ± 9.3	18	55
Stature (cm)	165.3 ± 8.7	147.5	187.3
Humerus (cm)	28.6 ± 2.4	23	36.1
Tibia (cm)	38.9 ± 2.6	33.5	46.5

Based on gender, participants’ age, stature, humerus length, and tibia length, with confidence interval (CI) at 95% is shown in Table 2.

**Table 2:** Gender distribution of age, stature, humerus length, and tibia length with CI at 95%. CI: confidence interval.

Variable	Male (M, n=82)				Female (F, n=82)			
	Mean ± SD	Minimum	Maximum	Confidence interval (CI) at (95%)	Mean ± SD	Minimum	Maximum	Confidence interval (CI) at (95%)
Age (year)	27.04 ± 9.73	18	55	24.90-29.18	26.18 ± 8.95	18	54	24.21-28.15
Stature (cm)	171.67 ± 5.67	158.7	187.3	170.42-172.92	159.07 ± 6.25	147.5	177	157.69-160.44
Humerus (cm)	29.53 ± 2.35	24.2	36.1	29.02-30.05	27.64 ± 2.03	23	34.3	27.19-28.09
Tibia (cm)	40.05 ± 2.49	33.9	46.5	39.50-40.60	37.77 ± 2.20	33.5	42	37.29-38.26

There was no significant difference in the age of the participants by gender at a 95% confidence interval (CI). Participants' stature, humerus length, and tibia length were significantly higher at 95% CI in males as compared to females (Table 2). Data were tested by the Shapiro-Wilk test for normality, and Pearson correlation was applied to see the correlation between age, lengths of humerus, and tibia with stature (Table 3).

**Table 3:** Pearson correlation coefficient of all variables with stature and p-values as per gender.

Variables	Male (M)		Female (F)		Combined (M+F)	
	Pearson's r	p-value	Pearson's r	p-value	Pearson's r	p-value
Age (year)	-0.305	<0.005	-0.113	<0.314	-0.108	<0.169
Humerus (cm)	0.719	<0.001	0.595	<0.001	0.701	<0.001
Tibia (cm)	0.809	<0.001	0.764	<0.001	0.801	<0.001

Correlations with stature

In males, stature correlated very strongly with tibia length (r = 0.809, p < 0.001) and strongly with humerus length (r = 0.719, p < 0.001); however, age showed a modest negative correlation (r = −0.305, p < 0.005). In females, stature also correlated strongly with tibia (r = 0.764, p < 0.001) and moderately with humerus (r = 0.595, p < 0.001) lengths, while the age-stature correlation was non-significant (r = −0.113, p = 0.314). When combining both genders, the correlations were similarly very strong for tibia (r = 0.801) and strong for humerus (r = 0.701), with a non-significant age association (r = −0.108, p = 0.169) (Table 3).

**Figure 1:**
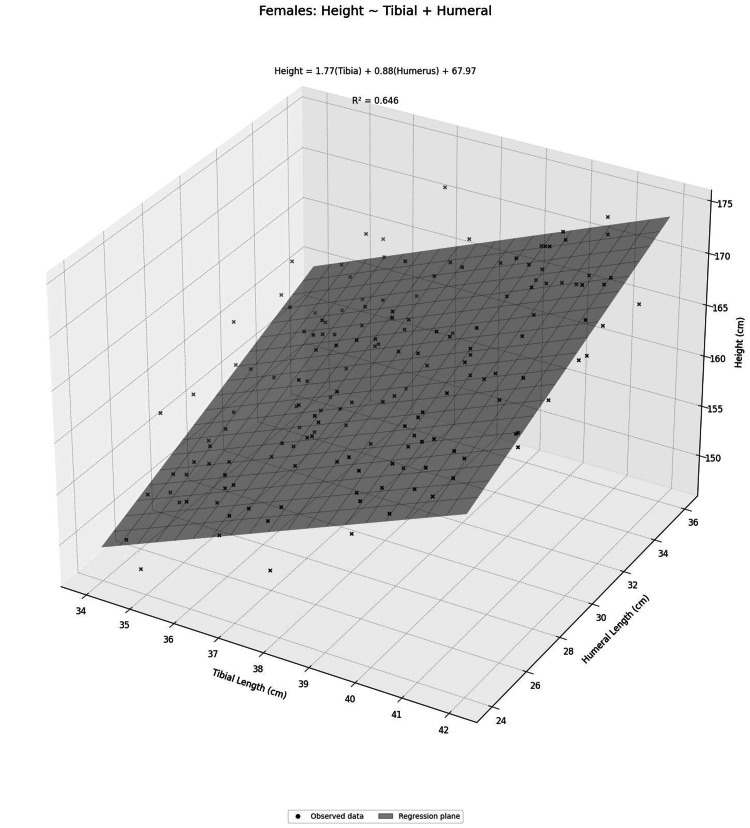
Scatter plot between height and combined tibia and humerus length in females.

Since age has shown an insignificant association with stature (Table 3), it was dropped. Thus, for predicting the stature, tibia and humerus lengths were utilized. A simple linear regression equation was constructed for each tibia and humerus as per gender separately, and multiple linear regression equation was constructed using both bones (predictors) as per gender (Table 4; Scatter plots: Figures 1-6 in Supplemental Material).

**Table 4:** Prediction of stature using tibia length and humerus length, and combined tibia and humerus as per gender. In male, the SEE of 3.9 in humerus, 3.3 in tibia, and 2.8 in combined model indicate that on average the predicted values differ from observed values by 3, 3, and 2 units, respectively, in the original measurement scale of dependent variables. The RMSE of 3.9 in humerus, 3.3 in tibia, and 2.8 in combined model indicate that models predicted values deviate from the observed values by 3.9, 3.3, and 2.8 on average. The MAE of 3.2 in humerus, 2.7 in tibia, and 2.3 in combined model indicate that on average the predicted values differ from observed values by 3.2, 2.7, and 2.3. In female, the SEE of 5.05 in humerus, 4.06 in tibia, and 3.7 in combined model indicate that on average the predicted values differ from observed values by 5, 4, and 3 units, respectively, in the original measurement scale of dependent variables. The RMSE of 4.9 in humerus, 4.01 in tibia, and 3.6 in combined model indicate that models predicted values deviate from the observed values by 4.9, 4.01, and 3.6 on average. The MAE of 4.1 in humerus, 3.2 in tibia, and 2.9 in combined model indicate that on average the predicted values differ from observed values by 4.1, 3.2 and 2.9 on average. Y: stature; X: variable; SEE: standard error of estimate; RMSE: root mean square error; MAE: mean absolute error.

Gender	Variables	Regression formula	r^2^	Adjusted r^2^	p-value	SEE	RMSE	MAE
Male	Humerus (cm)	Y = 120.51 + 1.73 X	0.517	0.511	<0.001	3.969	3.92	3.201
	Tibia (cm)	Y = 98.07 + 1.84 X	0.657	0.65	<0.001	3.357	3.316	2.745
	Combined (cm)	Y = 91.32 + 1.34 (tibia) + 0.90 (humerus)	0.747	0.74	<0.001	2.88	2.827	2.304
Female	Humerus (cm)	Y = 108.51 + 1.83 X	0.354	0.346	<0.001	5.058	4.996	4.132
	Tibia (cm)	Y = 77.08 + 2.17 X	0.584	0.578	<0.001	4.062	4.012	3.226
	Combined (cm)	Y = 67.97 + 1.77 (tibia) + 0.88 (humerus)	0.646	0.637	<0.001	3.734	3.665	2.903

The equation for stature estimation was derived in both males and females using humerus and tibia lengths individually, and the combined length of both bones (Table 4). Combined bones came out as the best predictor of stature in both male and female (r^2^=0.74, r^2^=0.64). The tibia alone performed well (M: r^2^=0.65 and F: r^2^=0.58) as compared to humerus (M: r^2^=0.51 and F: r^2^=0.35) in both genders (Table 4).

R^2^ represents the proportion of variance that can be explained by the regression model. In males, the regression equation in combined both bones is Y (stature) = 91.32 + 1.34 (tibia) + 0.90 (humerus) (r^2^=0.74), which explains 74% of the variability. In females, the regression equation in combined both bones is Y (stature) = 67.97 + 1.77 (tibia) + 0.88 (humerus) (r^2^=0.64), which explains 64% of the variability. An analysis model was also run with combining both genders into one data pool, and it also showed combined both bones as the best predictor (r^2^=0.70) as compared to humerus (r^2^=0.49) and tibia (r^2^=0.64) (Table 5).

**Table 5:** Linear regression formula for whole study population. The SEE of 6.2 in humerus, 5.2 in tibia, and 4.7 in combined model indicate that on average the predicted values differ from observed values by 6.2, 5.2, and 4.7 units, respectively, in the original measurement scale of dependent variables. The RMSE of 6.1 in humerus, 5.1 in tibia, and 4.6 in combined model indicate that models predicted values deviate from the observed values by 6.1, 5.1 and 4.6 on average. The MAE of 5.1 in humerus, 4.1 in tibia, and 3.8 in combined model indicate that on average the predicted values differ from observed values by 5.1, 4.1, and 3.8 on average. Y: stature; X: variable; SEE: standard error of estimate; RMSE: root mean square error; MAE: mean absolute error.

Variable	Regression formula	r^2^	Adjusted r^2^	p-value	SEE	RMSE	MAE
Humerus (cm)	Y = 92.66 + 2.54 X	0.49	0.487	<0.001	6.215	6.177	5.141
Tibia (cm)	Y = 61.77 + 2.66 X	0.64	0.638	<0.001	5.22	5.188	4.184
Combined (cm)	Y = 54.19 + 1.98 (tibia) +1.19 (humerus)	0.707	0.703	<0.001	4.727	4.684	3.825

## Discussion

This study presents contemporary, gender-based, and combined male and female pooled regression equations for stature estimation in the North Indian adult population using percutaneous humerus and tibia length measurements. In this population, in both genders, the tibia has emerged as the better single predictor of stature estimation when it comes to single long bones, but the combined bone model provided higher accuracy in predicting stature.

In this study, tibia length showed a stronger association with stature, as also observed in previous studies [5,9,10], which may be due to more contribution of long bones in the lower limb to stature as compared to upper limb bones [5]. In most populations, the tibia shows a correlation coefficient ranging from r = 0.85 to 0.95 with living stature, irrespective of gender [3,9,10]. In this study, the correlation found in males and females was r = 0.809 and 0.764, respectively, as compared to other Indian Oriya population [5] (M, r = 0.95; F, r = 0.93) with tibia bone, which reflects the diversity of the studied population as the participants belonged to different regions of India.

Stature estimation from the humerus has been demonstrated in different populations, and being a long bone of the upper limb, it has reliably shown a moderate to very strong correlation with stature [8,11,12,16]. This study showed comparable results in both genders (M, r = 0.71; F, r = 0.59) as seen in previous studies (M, r = 0.66-0.85; F, r = 0.50-0.79) with living stature [8,11,12,16], indicating variations in the different sets of populations and a lesser but positive correlation to stature as compared to the tibia.

In a Japanese adult sample measured by radiography, the tibia and femur were found to be strong stature predictors (r = 0.82-0.89) as compared to the humerus (r = 0.68-0.70) [8]. Another study derived multiple equations from using single long bones and also concluded that the tibia (r = 0.855) has the highest positive correlation when it comes to single long bones for male [4], emphasizing the more contribution of long bones of the lower limb in stature as compared to upper limb bones. 

Studies using a combination of multiple different long bones for stature estimation have been done across different groups of population and have proved to be a better predictor of stature as compared to a single long bone approach [2-4,7,8,13]. Our results are consistent with the South Indian study, which provided correlations using different combined long bones (femur and tibia) rather than using each bone separately [13]. An Indo-Mauritius population study using two long bones (tibia and ulna) and a Japanese study using three long bones (humerus, femur, and tibia) observed a higher coefficient of correlation [6,8]. The results indicate that dual-bone measurements capture complementary variation in body proportions, and a combination of long bones is more accurate than single-bone approaches [13].

This study highlights and addresses the gap by generating updated equations in (i) a gender-balanced adult sample, (ii) with two long bones to address complementary variation in body proportions in the North Indian population by using standardized and widely validated, percutaneous defined bony landmarks in long bones of the limbs. The main limitations are (i) the use of percutaneous landmarks that include soft tissue-related measurement variability and are not directly interchangeable with radiographic or osteometric measures; (ii) non-use of radiological or osteometric methods due to ethical and feasibility constraints, and (iii) an upper age of 55 years, limiting extrapolation to older adults in whom stature loss accelerates and prediction intervals widen.

This study contributes up-to-date equations derived from a gender-balanced adult sample and with percutaneous landmarks, which may be relevant to clinical anthropometry, sports science, and forensic settings like identification of the deceased based on the availability of skeletal remains in different situations where radiographic or osteometric data are not available.

## Conclusions

This study derived gender-stratified as well as pooled regression equations for determining stature from single long bones (humerus and tibia), and both bones in combination. All the measurements showed statistically significant correlation, with the combined bone approach showing strong positive correlation in all the cases. The long bones used in this study allow for accurate stature estimation for studied population, enhancing the use of multi-bone estimation in forensic science, anatomy, and other investigative settings. Further research with combinations of other long bones and a larger sample size, including higher age groups along with diverse populations, could refine these findings and build upon the results of this study.

## Figures and Tables

**Figure 2:**
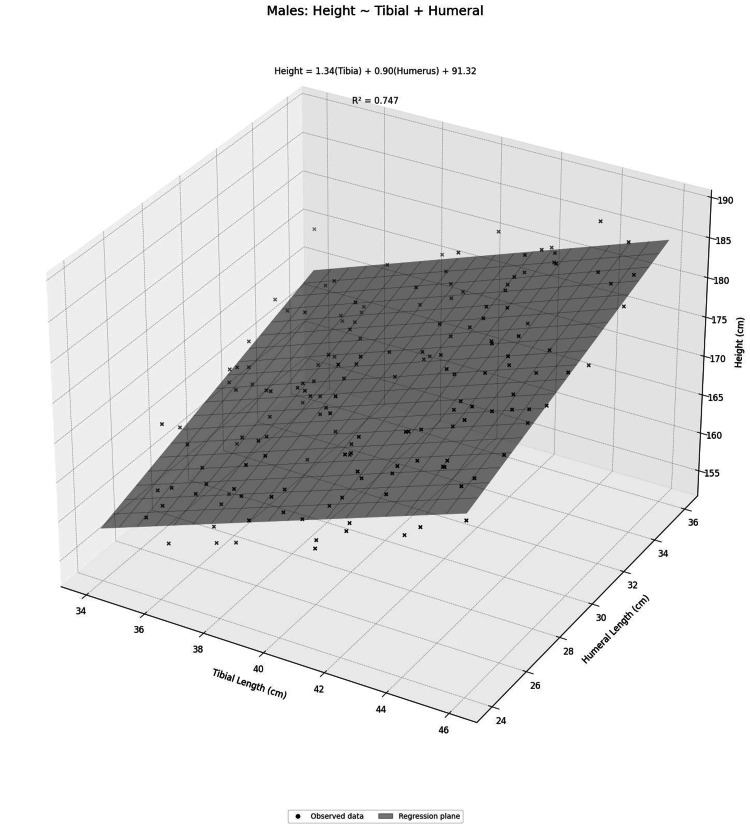
Scatter plot between height and combined tibia and humerus length in males.

**Figure 3:**
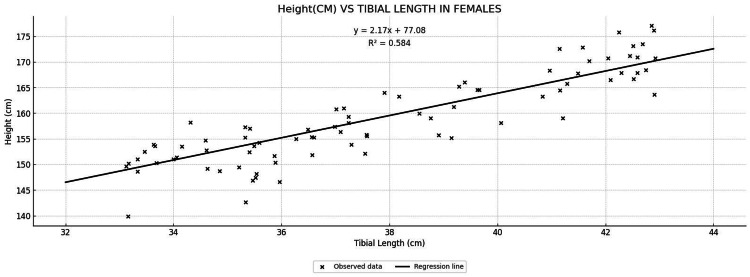
Scatter plot between height and tibia length in females.

**Figure 4:**
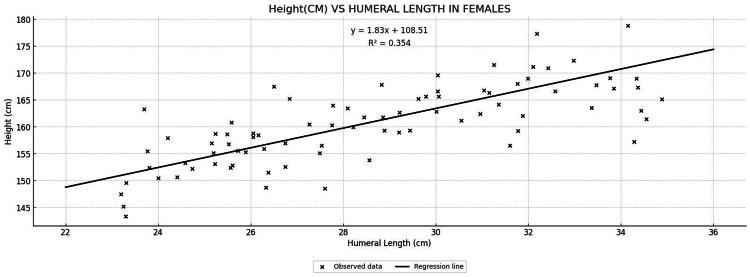
Scatter plot between height and humerus length in females.

**Figure 5:**
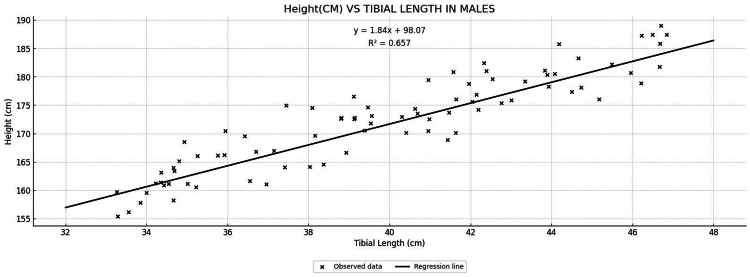
Scatter plot between height and tibia length in males.

**Figure 6:**
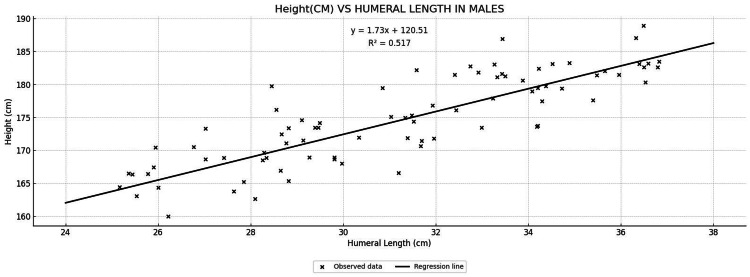
Scatter plot between height and humerus length in males.

## References

[1] Krishan K, Kanchan T, Menezes RG, Ghosh A (2012). Forensic anthropology casework-essential methodological considerations in stature estimation. J Forensic Nurs.

[2] Trotter M, Gleser GC (1952). Estimation of stature from long bones of American Whites and Negroes. Am J Phys Anthropol.

[3] Agnihotri AK, Kachhwaha S, Jowaheer V, Singh AP (2009). Estimating stature from percutaneous length of tibia and ulna in Indo-Mauritian population. Forensic Sci Int.

[4] Menéndez Garmendia A, Sánchez-Mejorada G, Gómez-Valdés JA (2018). Stature estimation formulae for Mexican contemporary population: a sample based study of long bones. J Forensic Leg Med.

[5] Mohanty NK (1998). Prediction of height from percutaneous tibial length amongst Oriya population. Forensic Sci Int.

[6] Didia BC, Nduka EC, Adele O (2009). Stature estimation formulae for Nigerians. J Forensic Sci.

[7] Kate BR, Mujumdar RD (1976). Stature estimation from femur and humerus by regression and autometry. Acta Anat (Basel).

[8] Hasegawa I, Uenishi K, Fukunaga T, Kimura R, Osawa M (2009). Stature estimation formulae from radiographically determined limb bone length in a modern Japanese population. Leg Med (Tokyo).

[9] Ghosh T, Konar S (2019). Estimation of stature from percutaneous length of tibia amongst Bengali population. Int J Res Med Sci.

[10] Mehta AA, Mehta AA, Gajbhiye VM, Verma Sarthak (2015). Correlation of percutaneous tibial length with body height and estimation of stature in living central India population. Int J Anat Res.

[11] Borkar Borkar, M. P (2017). Estimation of height from the length of humerus in western region of Maharashtra. Int J Res Med Sci.

[12] Prateek G, Shalini G, Anupama M, Chakravarthi KK (2013). Estimation of height using length of humerus in adult North Indian population-an anthropometric study. International Journal of Scientific Research.

[13] Tangirala R, Nagendra Prasad B, Umamaheswara Rao P, Balaji Singh M (2021). Estimation of ‘Stature’ from long bones of the lower limb — a cadaver based study. Indian J Forensic Med Toxicol.

[14] Green SB (1991). How many subjects does it take to do a regression analysis. Multivariate Behav Res.

[15] Wuensch KL (1996). [Review of Straightforward Statistics for the Behavioral Sciences, by J. D. Evans]. J Am Stat Assoc.

[16] Oghenemavwe LE, Agi CE (2022). Stature estimation from humeral length amongst Nigerians: a radiographic approach. World J Biol Pharm Health Sci.

